# Assessment of Accuracy of Spatial Object Localization by Means of Mono and Stereo Modes of Visual-to-Auditory Sensory Substitution in People with Visual Impairments (a Pilot Study)

**DOI:** 10.17691/stm2024.16.4.03

**Published:** 2024-08-30

**Authors:** A.S. Butorova, E.A. Koryukin, N.M. Khomenko, A.P. Sergeev

**Affiliations:** Junior Researcher; Institute of Industrial Ecology of the Ural Branch of the Russian Academy of Sciences, 20 S. Kovalevskoy St., Ekaterinburg, 620990, Russia; PhD Student; Ural Federal University named after the First President of Russia B.N. Yeltsin, 19 Mira St., Ekaterinburg, 620002, Russia; Research Assistant, Artificial Intelligence Laboratory; Ural Federal University named after the First President of Russia B.N. Yeltsin, 19 Mira St., Ekaterinburg, 620002, Russia; Student; Ural Federal University named after the First President of Russia B.N. Yeltsin, 19 Mira St., Ekaterinburg, 620002, Russia; Research Assistant, Artificial Intelligence Laboratory; Ural Federal University named after the First President of Russia B.N. Yeltsin, 19 Mira St., Ekaterinburg, 620002, Russia; PhD, Leading Researcher; Institute of Industrial Ecology of the Ural Branch of the Russian Academy of Sciences, 20 S. Kovalevskoy St., Ekaterinburg, 620990, Russia

**Keywords:** visual impairments, visual-to-auditory sensory substitution, sound representation

## Abstract

**The aim of the study** is to assess the accuracy of spatial object localization in mono and stereo modes of visual-to-auditory sensory substitution by means of the developed system tested on persons with normal or corrected-to-normal vision.

**Materials and Methods:**

A prototype of a visual-to-auditory sensory substitution device based on a video camera with two lenses was prepared. Software to convert the signal from a video camera into an audio signal in mono and stereo modes was developed.

To assess the developed system, an experimental study with 30 blindfolded sighted participants was conducted. 15 persons were tested in mono mode, 15 — in stereo mode. All persons were trained to use the visual-to-auditory sensory substitution system. During the experiment, participants were to locate a white plastic cube with dimensions of 4×4×4 cm^3^ on a working surface. The researcher placed the cube in one of 20 positions on the working surface in a pseudo-random order.

**Results:**

To assess the accuracy of the cube localization, deviations along the *X-* and *Y*-axes and absolute deviations were calculated. The general dynamics of localization accuracy was positive both in mono and stereo modes. Absolute deviation and *X*-axis deviation were significantly higher in stereo mode; there was no significant difference in *Y*-axis deviation between modes. On average, participants tended to underestimate the distance to the cube when it was on the left, right, or far side of the working surface, and overestimate the distance to the cube when it was on the near side of the working surface.

**Conclusion:**

Tests demonstrated that the accuracy of object localization in stereo mode can be improved by increasing the time for training the participants and by showing them more presentations. The results of the study can be used to develop assistive techniques for people with visual impairments, to manufacture medical equipment, and create brain-computer interfaces.

## Introduction

According to the World Health Organization, at least 2.2 billion people globally have various forms of visual impairment, including blindness [1]. The number of such persons is expected to increase significantly in the coming years due to demographic growth and the population aging. In Russia, according to statistics of the Ministry of Health, an average of 4.7 million ophthalmic diseases are registered annually. In 2022, 3.6 million ophthalmic diseases were diagnosed [2].

To improve the quality of life of people with visual impairments, various techniques are being developed; these techniques are to solve the problem of patients’ self-navigation. Currently, this is achieved by using both traditional approaches (guide dogs, Braille, tactile indicators) and modern invasive and non-invasive techniques.

Invasive techniques include implanting microchips directly into the retina or optic nerve [3]. Microchips can convert light signals into electrical impulses that are transmitted to the brain and interpreted as visual signals. Such techniques are associated with the risk of complications and implant failure and require a complex surgery.

Unlike invasive, non-invasive techniques do not require surgical intervention. Such techniques include transcranial magnetic stimulation [4], magnetic brain stimulation with nanoparticles [5], and sensory substitution [6].

Sensory substitution is a process by which the impaired function of an organ or sense is compensated by other organs or senses [7]. The process of sensory substitution of vision is conducted by means of two main types of devices: visual-to-tactile and visual-to-auditory. Visual-to-tactile devices convert camera image into a tactile signal, which is transmitted to various parts of the body [8]. Visual-to-auditory sensory substitution allows to transmit information about the external environment using sound signals.

One of the first visual-to-auditory sensory substitution systems, The vOICe, was developed by Meijer [9]. Using a specific algorithm, The vOICe system converts the signal from the video camera into an audio signal. The height of the object’s position in the camera field of view is encoded by an audio tone: the higher is the position of the object in the field of view, the higher is the tone of the sound. The brightness of an object is encoded by volume: the brighter (lighter) is the object, the louder is the sound.

The authors [10] mention 4 stages of formation of representation, which a person accepts when he is trained to perceive the space and objects using The vOICe sensory substitution system: diffuse perception, syncret, preconception, and concept. Due to the feedback mechanism, the trained persons — as time passes — identify objects around them from the sound, find the differences between sound patterns and shapes of different objects, and, finally, can attribute the sound of an unfamiliar object to a certain category.

In 1998, a prototype of another visual-to-auditory sensory substitution device was developed, it was called PSVA (Prosthesis for Substitution of Vision by Audition) [11]. The PSVA operating principle is that each pixel in the received image is associated with a sinusoidal audio frequency signal. The totality of these sinusoidal signals is reproduced by means of headphones. The vOICe and PSVA convert black and white images and do not convey color information. EyeMusic [12] and SeeColOr [13] are systems that convert color images into sound. In [14], the authors used artificial intelligence methods to preprocess the signal from a video camera.

Application of visual-to-auditory sensory substitution devices is associated with a number of challenges. One of them is a long-term training of users of visual-to-auditory sensory substitution devices: from several months to several years [15–17]. Low availability and affordability of visual-to-auditory sensory substitution devices affect their use by people with visual impairments. Due to their limited production and high cost, the devices are inferior to more affordable and convenient devices. Moreover, sensory substitution devices are tested primarily in laboratory conditions, which are far from the real-life environment. This may lead to incomplete or distorted information about the actual performance of visual-to-auditory sensory substitution devices [18]. Finally, users of such devices experience sensory overload — a condition when the brain receives too many sensations from external stimuli, which leads to tiredness and, thus a decreased concentration [18, 19]. In [20], a visual-to-auditory sensory substitution system used a video camera with two lenses to provide the user with an audio representation of the world around. The system was used to navigate in various scenarios, including real-life environments.

The authors of this study assumed that stereo mode in the visual-to-auditory sensory substitution system, which converts the signal from a video camera with two lenses, provided for stimulation of human binocular vision and thus increased the accuracy of spatial localization of an object. The following hypothesis was given: the use of stereo mode in the visual-to-auditory sensory substitution system would increase the accuracy of spatial object localization compared to mono mode.

**The aim of the study** is to assess the accuracy of spatial object localization in mono and stereo modes of visual-to-auditory sensory substitution by testing the developed system on persons with normal or corrected-to-normal vision.

## Materials and Methods

### Device prototype and experiment setup

**Figure 1.**
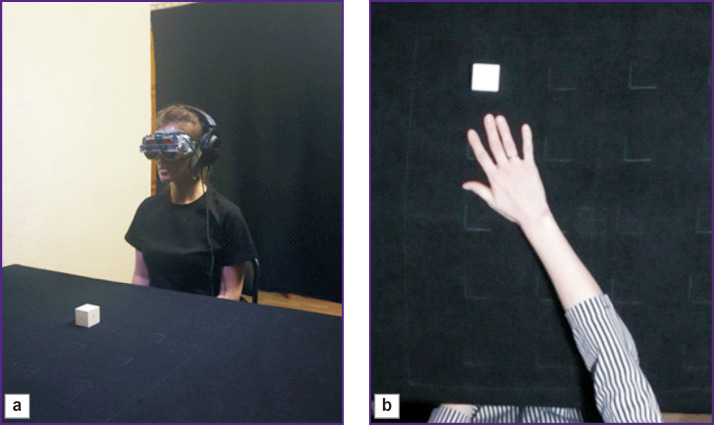
Experiment setup: (a) the prototype of the visual-to-auditory sensory substitution device; (b) the white cube on the black table surface

The prototype of the visual-to-auditory sensory substitution device is made on the basis of glasses and a digital video camera with two lenses (Figure 1 (a)). The signal from the video camera is transmitted through a USB port to a laptop. This signal is converted into sound using the original software. The audio signal is read from the laptop and played back through wired headphones.

This device is intended for persons with visual impairments of Categories 3–5 according to the ICD-10 classification of visual impairment severities (H54 Visual impairment including blindness) [21]. These categories include persons with visual impairments who require assistance with daily activities, use a white cane, guide dog, or guide person to navigate.

The experiment setup consists of a table covered with black cloth (Figure 1 (b)). It is surrounded by a black fabric screen on each side to eliminate glare and noise. The working surface dimensions are 600×600 mm^2^. Local lighting is installed for the working surface; there is no basic lighting in the room. The experiment is video recorded by a video camera installed above the working surface so that its focal plane is parallel to the table surface. A white plastic cube with dimensions of 4×4×4 cm^3^ is used as the manipulated object.

### Software

The developed software receives an image from a digital video camera with two lenses, which create two separate frames on two separate matrices. The software captures images from two lenses and changes the image color space from RGB to black-and-white. To capture images from a video camera, the authors used the Python OpenCV library; images conversion into sound was implemented in the C language.

**Figure 2.**
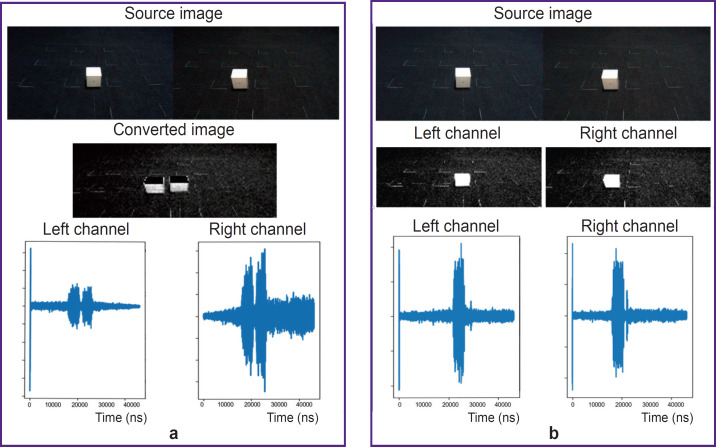
Mono (a) and stereo modes (b)

The software can work in two modes: mono or stereo (Figure 2). In mono mode, The vOICe algorithm is used [9]. Stereo mode was developed by the authors. In mono mode, two source images are merged into one in accordance with the average brightness value of the source images. The image size is reduced to 176× 64 pixels, and the brightness and contrast of the image are changed according to the preset rules. In stereo mode, the software performs the same transformations for each of the two source images separately.

Then, in mono mode, the program splits the image into 1-pixel wide rows and columns and calculates the audio signal value for each pixel at the row-column intersection. Two parameters of the sound signal are calculated: amplitude and frequency. The amplitude of the sound signal is calculated in proportion to the pixel brightness: the higher is the brightness value, the greater is the amplitude of the sound signal. The frequency of the sound signal is calculated in proportion to the pixel position coordinate along the vertical axis: the greater is the coordinate, the higher is the sound frequency. Then, the software sums the audio signal values across columns of pixels sequentially from left to right and writes them to an audio file. When playing the audio file, the right channel volume is set higher for the first half of the file, and the left channel volume is set higher for the second half of the file.

In stereo mode, the software splits and calculates the parameters of the audio signal similar to mono mode, but for two images separately. Stereo mode differs from mono in the fact that the software writes signal values from the left frame to the left channel of the audio file, and signal values from the right frame to the right channel of the audio file.

### Experimental study

To assess the developed system performance, an experimental study was conducted in accordance with the ethical principles of the Declaration of Helsinki (2013). Permission was received from the Ethics Commission of the Ural Federal University named after the First President of Russia B.N. Yeltsin (extract from the Minutes of Meeting No.02 dated March 10, 2023).

All participants signed informed consent to participate in the study and consent to study data processing (participant data was anonymized). Participation in the experiment was voluntary; each person could refuse to participate at any moment, no explanation was required. Participants had normal or corrected-to-normal vision and reported no history of neurological or psychiatric diseases. None of the participants had previous experience of using sensory substitution devices to locate objects or explore a 3D environment.

30 persons aged from 18 to 51 years participated in the experiment. Two groups were formed: 15 people were tested in mono mode, and 15 — in stereo mode. The groups were balanced by gender and age: 15 men with the mean age of 30.2 years and 15 women with the mean age of 30.7 years.

During the experiment, the participants were blindfolded, so none of them could see the setup until the end of the experiment. Calibration was performed before the experiment: the researcher placed the cube in the center of the working surface of the table, the participants listened to how the cube sounded and covered it with a palm. After that, the researcher placed the cube at its upper and lower positions on the working surface to give participants an idea of the working surface dimensions.

All participants were trained to work with the visual-to-auditory sensory substitution system. The researcher thoroughly explained the rules for transcoding images into sound and asked the participants to perform training tasks: localize and grasp a cube in the central, lower right, and upper left positions on the working surface. To determine the cube location, the participants had to center the camera so that the cube was directly in front of them. The centered cube sounded with a medium tone in the middle of the audio track. Instructions on locating the cube were taken from the Russian version of The vOICe Training Manual without significant changes: “For vertical alignment, you need to tilt your head up and down until the object beep is at medium pitch (neither high nor low). Next, while maintaining this pitch, you turn your head left and right until the beep sounds half a second after the start of each soundscape, that is, in the horizontal middle of the default one-second duration of each soundscape scan. The direction from which the object sound seems to come will then also be straight ahead and not to the left or right. Then you can reach out and grab the object, imagining that it is in the direction where your nose is pointing” [22]. After training, the persons proceeded with testing.

During the test, the white cube was randomly placed in one of 20 positions on the working surface of the table. Each participant was presented with the same sequence of cube positions. The person sat on a chair in front of the table and explored the setup by tilting the head and body. It was impossible to get up and move around the room. After 10 presentations of the cube, all participants were offered a 10-minute break, but they could refuse the break and continue the test.

To assess the accuracy of the cube location, deviations along the *X-* and *Y*-axes with the corresponding sign and absolute deviations were calculated. For this purpose, central points were marked on the cube edges and the participants’ hand backs. Deviations along the *X-* and *Y*-axes were calculated as the difference between the center point of the top edge of the cube and the point marked with a black marker on the participant’s hand back. Absolute deviations *del* were calculated using the following formula:

del=delX2+delY2,

where *delX* is the deviation along the *X*-axis; *delY* is the deviation along the *Y*-axis.

**Statistical data processing** was performed in the Statistica software package (v. 12). The statistical significance of differences in the results of the two experimental groups was assessed using the non-parametric Mann–Whitney test, as differences between independent groups were considered.

In the mono mode group, the absolute deviation was 125.9±98.1 mm; in the stereo mode group — 173.6±113.2 mm.

## Results

### General dynamics of cube localization accuracy

**Figure 3.**
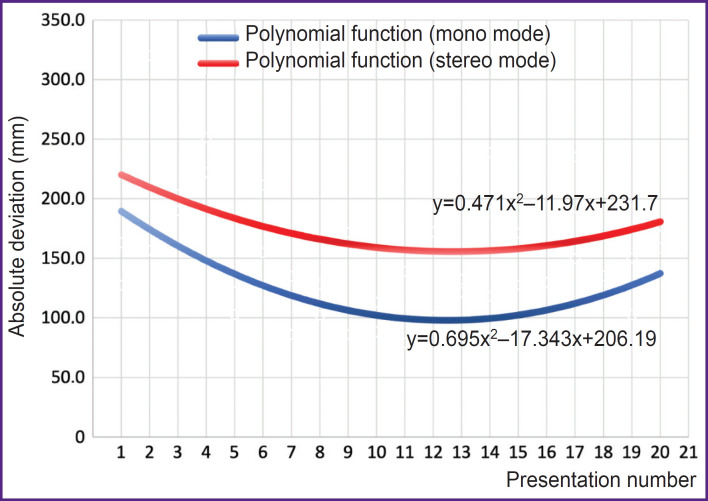
General dynamics of the cube localization accuracy

The dynamics values were positive in both mono and stereo modes. The observed absolute deviation decreased from the 1^st^ to the 12^th^–14^th^ presentation. Then, from the 14^th^–15^th^ to the 20^th^ presentation an increase in the absolute deviation was seen, but the initial absolute deviations were not achieved (Figure 3).

### Absolute deviations

The values of absolute deviations were statistically significantly higher in stereo mode (Mann– Whitney test, p<0.05). The deviation along the *X*-axis was also statistically significantly higher in stereo mode (Mann– Whitney test, p=0.03). There was no statistically significant difference in *Y*-axis deviation between modes (Mann–Whitney test, p=0.38).

**Figure 4.**
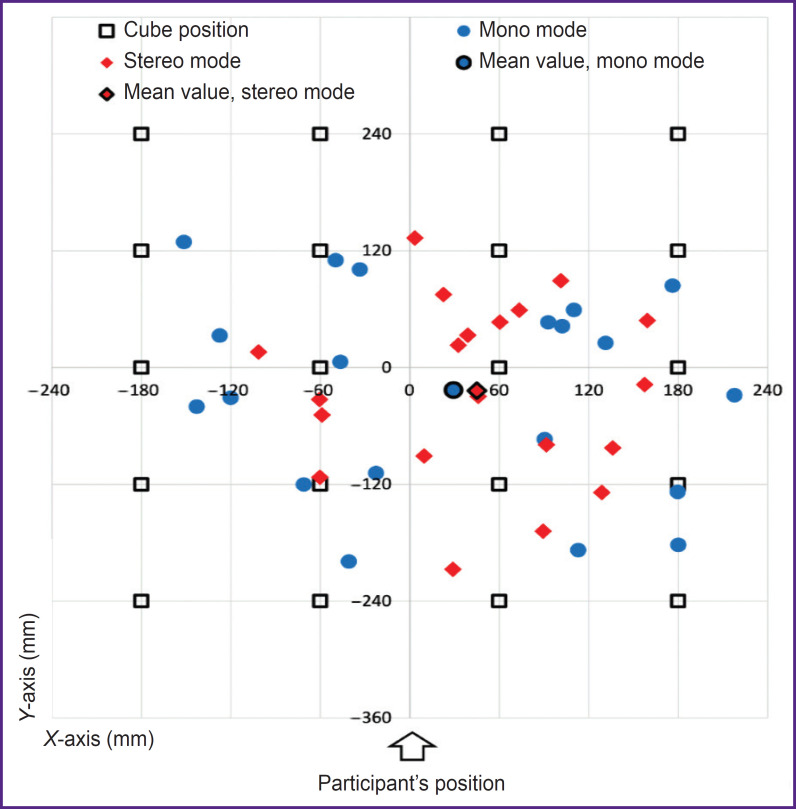
Absolute deviations of object localization accuracy values

In both modes, participants tended to bias toward the center of the work surface in both the *X*- and *Y*-axes. On average, participants tended to shift the *X*-axis to the right, with the *X*-shift being slightly higher in stereo mode. On average, participants underestimated the distance to the cube along the *Y*-axis, and underestimation was approximately comparable in mono and stereo modes (Figure 4).

### Deviations along the X- and Y-axes

**Figure 5.**
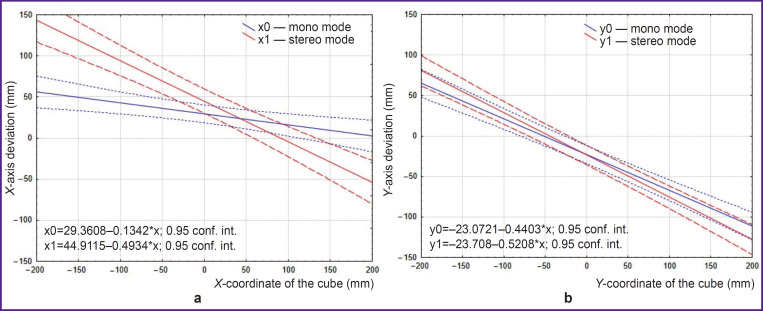
Deviations along the *X*-axis (a) and *Y*-axis (b)

When the cube was on the left side of the working surface (Figure 4, area of negative values along the *X*-axis), participants tended to underestimate the distance to the cube. Moreover, the further to the left the cube was located, the more the persons underestimated the distance to it. The underestimation of the distance to the cube in stereo mode was higher than in mono mode. When the cube was on the right side of the working surface (Figure 4, area of positive values along the *X*-axis), the participants also underestimated the distance to the cube. Here, the further to the right the cube was located, the more the persons underestimated the distance to the cube (Figure 5 (a)).

When the cube was in the lower part of the working surface (Figure 4, area of negative values along the *Y*-axis), the participants tended to overestimate the distance to the cube; and when it was in the upper part of the working surface (Figure 4, area of positive values along the *Y*-axis), the participants tended to underestimate the distance to the cube. At that, overestimation of the distance to the cube located in the lower part of the working surface was 2 times less than underestimation of the distance to the cube located in the upper part of the working surface. Approximately comparable deviations along the *Y*-axis were seen for both modes (Figure 5 (b)).

## Discussion

Study of the sensory substitution system performance involves several stages, the first of which is experimental testing of the developed system on people with normal or corrected-to-normal vision. Here, the accuracy of spatial localization of the cube in mono and stereo modes of visual-to-auditory sensory substitution was assessed by testing on persons with normal or corrected-to-normal vision.

The general dynamics of cube localization accuracy was positive both in mono and stereo modes. The observed increase in absolute deviation from the 14^th^–15^th^ to the 20^th^ presentation may indicate sensory overload experienced by the participants, which led to a decrease in the accuracy of the cube localization. This effect is consistent with results of other studies [23, 24]. However, a better solution of sensory overload requires a modification of the visual-to-auditory sensory substitution algorithm. Transformation and transmission of each information package captured by the video camera creates an uncomfortable stimulation. Therefore, development of algorithms for visual-to-auditory sensory substitution raises a fundamental issue related to the following: which amount of data from the video camera field of view which is to be converted into sound and whether this information is to be transmitted as is or significantly preprocessed and simplified when converted to sound.

The authors advanced a hypothesis that the use of stereo mode in the visual-to-auditory sensory substitution system would increase the accuracy of spatial localization of a cube. However, the absolute deviation and the *X*-axis deviation were statistically significantly higher in stereo mode. On average, participants estimated depth distance to the object with equal accuracy in both modes, but in stereo mode they significantly underestimated the width distance to the right and left. Hence, stereo mode did not demonstrate an increase in accuracy, which did not allow to confirm the hypothesis. The result obtained, however, may indicate that the training time is insufficient to successfully master using stereo mode.

In both modes participants tended to shift the object to the center of the working surface in both the *X-* and *Y*-axes. This result is consistent with the authors’ previous work on depth perception in visual-to-auditory sensory substitution [19]. Such a systematic tendency to shift the object to the right along the *X*-axis may point to the functional brain asymmetry of participants in case of an object localization task [25].

The results obtained allow to specify further areas of research:

1. Assessment of subjective sensory overload of persons in mono and stereo modes of visual-to-auditory sensory substitution by means of questionnaires, for example, the NASA-TLX load index [26].
2. Assessment of the accuracy of an object spatial localization in stereo mode with more trial presentations.
3. Experimental testing of mono and stereo modes in a real-life environment and modes adjustment taking into account the environment peculiarities.
4. Testing of the developed modes on persons with impaired vision.

## Conclusion

The study offered mono and stereo modes of visual-to-auditory sensory substitution and tested them on persons with normal or corrected-to normal vision. The authors prepared a prototype of a visual-to-auditory sensory substitution device based on a video camera with two lenses and developed software to convert the signal from the video camera into sound in mono and stereo modes.

The study results allow to prove the perspectives for development of assistive techniques for people with visual impairments, as well as brain-computer interfaces.
